# Blastomycosis in New York State. A Question1Read at the forty-first annual meeting of the Medical Association of Central New York, held at Buffalo, October 27, 1908.

**Published:** 1909-06

**Authors:** Henry Clay Baum

**Affiliations:** Syracuse N. Y.


### Blastomycosis in New York State. A Question.[1]

By HENRY CLAY BAUM, M. D., Syracuse N. Y.

SINCE attention has been directed to this disease, some cases crop up from time to time, that seem to compel the diagnosis of blastomycosis: this, too, in spite of the fact that we are not supposed to find the disease in New York state. The excuse for this article appearing on the program is the hope of determining whether one experience is exceptional, or if in truth

1. Read at the forty-first annual meeting of the Medical Association of Central New York, held at Buffalo, October 27, 1908.

the disease is really not so rare among us, but classed under other names by physicians seeing only occasionally cases of the rarer forms of skin diseases.

I did not recognise the first case and should not have been able to diagnose it, I fear. It was unmistakably an infection of a severe type, and through a suggestion received in a letter from Dr. J. C. Johnston, I was finally able to identify it. Dr. James Nevins Hyde of Chicago, has given this disease the intelligent study characteristic of his work, and furnishes a description, than which there is none clearer and more helpful, from which to quote:

Blastomycosis is a chronic, inflammatory, infectious disease, characterised by the appearance on the skin of a papule or papulo-pustule which becomes crusted and extends peripherally to form a sharply outlined, elevated verrucous patch, situated upon a pus infiltrated base and presenting a characteristic, abruptly sloping border in which are seen minute, deeply seated abscesses. Blastomycetes are found in the seropurulent contents of the abscesses, from which both budding and mycelial forms of the organism have been obtained in pure culture.

One is reluctant to stop here, for Dr. Hyde's description of the symptomatology and pathology is most instructive. Many others have contributed to the literature and scientific knowledge of this subject. Dr. Gilchrist, of Johns Hopkins, should be named, for having demonstrated the responsible organisms in a case which Duhring considered a scrofuloderm and for his repeated studies and contributions.

Many others are entitled to share the credit for bringing to light the facts concerning blastomycosis, but that is not germain to this paper. My first case occurred in the person of a colleague from a neighboring town. Although no longer practising medicine, he was able to give a very clear record of his case. The first lesion came on the dorsum of his right hand and gradually extended to the wrist and beyond, proximally, and to the first phalanx of each digit. A second lesion appeared on the right cheek, opposite the upper lip. A third lesion, size of a silver dollar, came back of his left ear, just in the hairy scalp.

Ultimately this lesion recovered so fully that even the hair grew again and no scarring is to be seen. The first lesion left his hand atrophic and weak and the scar is tense, glistening and thin, with dilated capillaries running through it, as in the cicatrices of burns. The scar of the lesion of the right cheek is much less conspicuous and might easily escape notice. Still a fourth lesion developed later, on the left ala nasi. and is not yet extinct, though an atrophic scar gives a pinched and wasted appearance to the healed portion while the characters of blastomycosis are found at parts of the extending border where the disease persists.

At one time there developed a somewhat generalised adenitis; and from the affected hand to the corresponding axilla was a chain of nodes of the size of pigeons' eggs, of the violaceous color of erythema nodosum, which were both tender and spontaneously painful. At this time the patient was marasmic, with sluggish intellectuation and seemed about to die. But he rallied under energetic treatment and is now able to attend to his occupations, although far from being a well man. Dr. Hyde saw this case and concurred in the diagnosis although none connected with the case had been able to find the organisms. Hyde also failed, after repeated efforts, without influencing his opinion as to the nature of the disease. Later on this case was presented at the Niagara Falls meeting of the American Dermatological Society and again identified by Dr. Hyde and others present familiar with the disease as blastomycosis. Fortunately, in the mean time, Dr. Adelaide Dutcher found the organisms in the secretion in the miliary abscesses and obtained pure cultures in both forms. Although using no different technic, but by the same identical methods which for many months had brought only negative results. The smears and cultures were shown with the case, which was the means of convincing some of those present, who, by the way, confessed that they had not previously seen a case of blastomycosis.

Case II. Miss D., age 10, lesion on dorsal aspect of right leg. Recovered under *x*-rays and the iodide of potash, but relapsed some years later. At the time of her relapse she was living in another town. I have not seen her, she being treated by a colleague in her own town, but have been informed that the lesions were the same and recurred in the scar of the previous disease.

Case III. Mrs. W., age 55, lesion on dorsum of right hand. Cleared up promptly under vigorous high frequency treatments, by vacuum electrode, and remains well.

Case IV. Mrs. K., age 50, multiple lesions on right leg and thigh, with some few on left leg also, and two on the face. Recovered under local dressings of antiseptics and especially did well with an acidulated peptic solution applied continuously. Salicylate of mercury by mouth. No recurrence.

Case V. Mr. H., age 18, lesion on dorsal aspect of right leg. Healed finally after a great variety of treatments, including *x*-rays, high frequency treatments, various local applications, of which the most satisfactory was equal parts of ichthyol, lanolin and vaseline. Internally, iodide of potash in rather large dosage. There was no real gain in this case until the iodide was pushed. There has been no relapse.

Since that time several similar cases have been found in and about Syracuse apparently clinically identical with the first.

Some other cases have been left out of this group because of irregular and atypic features and because we have been unable to demonstrate the organism. Yet it is as difficult to exclude them as to classify them elsewhere. Writers on blastimycosis are apt to declare that the organisms are easily found. Usually, perhaps this is true. Some observers have stated that they only found the organisms after patient and repeated attempts. Certainly it was only after months of failures that the organisms were found in the first case here reported: which, if the asserted easy demonstration is a constant and dependable character should have excluded it from its proper classification.

In conclusion, it seems possible that these cases are not so infrequent in New York state as they have been thought. That even as Duhring thought his case a scrofuloderm, so may others regard an occasional case. Or, it might be confused with a syphilide, tuberculide, ringworm or even an eczema. In differentiating, it should be kept in mind that the organisms may prove elusive.

809 University Block.

